# Microscopical Note

**Published:** 1897-04

**Authors:** 


### MICROSCOPICAL NOTE.

At a small town near Pittsburg, Alex. Killen was charged with robbing and murdering a woman who had owned a jewelry store. The culprit had broken a window and scraped the jewelry into a yellow satchel. In the haste some glass was included.

After Killen's arrest, such a satchel was found on his premises and some small pieces of glass found in it. At the trial, the District Attorney laid them on a sheet of paper and passed them to the jury. This was not satisfactory—a powerful microscope was brought in and each juror examined the bits of glass. A glass worker on the jury was satisfied that the bits were from window glass and not from a bottle which Killen said had been broken in the satchel. The other jurors accepted his suggestions and convicted Killen of Murder in the first degree—circumstantial evidence adduced by the use of a microscope.—*Microscopical Journal.*

---

TO REMOVE A FOREIGN BODY FROM THE NOSE, URETHRA, ETC.—Beugnies describes a simple arrangement with which he removes foreign bodies from small passages. A hole is bored in the end of a probe and a thread fastened in it. This is then introduced into the passage and carefully pushed past the foreign body. The string then held in one hand and the probe in the other, the little whip thus forms a loop with which the foreign body is easily withdrawn.—*Gaz. Med. de Liege, Univers. Med. Jour.*

